# Enhanced genome editing in rice using single transcript unit CRISPR‐*Lb*Cpf1 systems

**DOI:** 10.1111/pbi.13028

**Published:** 2018-11-19

**Authors:** Rongfang Xu, Ruiying Qin, Hao Li, Juan Li, Jianbo Yang, Pengcheng Wei

**Affiliations:** ^1^ Key Laboratory of Rice Genetic Breeding of Anhui Province Rice Research Institute Anhui Academy of Agricultural Sciences Hefei China

**Keywords:** single transcript unit, CRISPR, LbCpf1, genome editing

A variety of sequence‐specific nucleases (SSNs) have been successfully used for plant genome editing. Clustered regularly interspaced short palindromic repeat (CRISPR)‐CRISPR‐associated (Cas) systems, which were developed from microbial adaptive immune systems, are preferred SSN tools due to their simplicity and versatility. The majority of plant genome editing studies have exploited a CRISPR‐Cas9 system from *Streptococcus pyogenes* (*Sp*Cas9) to achieve site‐specific mutagenesis and perform fragment insertion or replacement in organisms ranging from algae to higher plants. A newly identified Cas endonuclease named Cpf1 is also considered a promising genome editing tool (Zetsche *et al*., 2015). A Cpf1 ortholog from *Lachnospiraceae bacterium ND2006* (*Lb*Cpf1) have been identified that can be programmed to induce site‐specific mutagenesis in eukaryotic cells (Zetsche *et al*., 2015). Some features of the Cpf1 system differ from those of Cas9, which expand the application of genome editing (Zetsche *et al*., 2015). Cpf1 recognizes a T‐rich protospacer adjacent motif (PAM) located 5′ of the target region in contrast to the 3′‐G‐rich PAM favoured by Cas9. Cpf1 performs staggered DNA cleavage distal to the PAM site, whereas Cas9 makes blunt cuts proximal to the PAM site. Moreover, Cpf1 requires only a single CRISPR RNA (crRNA) and processes its own crRNA array, which facilitates multiplexed genome editing (Wang *et al*., 2017). Several groups, including ours, have recently reported the successful application of the CRISPR‐*Lb*Cpf1 system in plant species such as rice, Arabidopsis, soybean and tobacco (Tang *et al*., 2017; Wang *et al*., 2017; Xu *et al*., 2017). However, the efficiency was variable in these systems.

Coexpressing sgRNA and SpCas9 separated by ribozyme (RZ) cleavage sites in a single transcriptional unit (STU) provided a conditional, simple and highly active system for plant genome editing (Tang *et al*., 2016). Cpf1 contains intrinsic crRNA processing activity, and previous reports indicated that Cpf1 can generate efficient multiplexed targeted mutagenesis combined with a directly expressed crRNA array (Wang *et al*., 2017). It is reasonable to presume that active crRNAs can be produced from a Pol II transcript. Therefore, we modified our previously reported *Lb*Cpf1 vector by integrating crRNA at the 3′ end of the *Cpf1* transcript and expressing from the single constitutive promoter (designated as STU vector). Furthermore, a poly‐A linker to stabilize *Lb*Cpf1 translation (Tang *et al*., 2016) and a tRNA sequence for precise crRNA expression (Ding *et al*., 2018) were separately or jointly applied to enhance editing performance (Figure 1a). Two rice genome targets located in the *OsPDS* and *OsGS3* gene were selected to test the activity of vectors. Five constructs of each site were stably transformed in parallel rice (*Japonica*. Nipponbare) transgene experiments. The targeted mutations were confirmed by Sanger sequencing followed by HRM analyses or Hi‐tom NGS detection (SRP158309). The highest mutation frequencies of both targets were detected in the plants carrying the STU‐poly‐A vector. The mutagenesis efficiency reached up to 77.8% and 91.7% for OsPDS and OsGS3, which is 3.62‐ and 2.09‐fold higher, respectively, than the original Pol III promoter vector (Figure 1b). The biallelic mutation rates were also increased by 3.42‐ and 2.23‐fold with the STU‐poly‐A vector. These results suggest that the crRNA might excise from chimeric mRNA and improved targeted mutation activity. The low mutation frequency of our previous *Lb*Cpf1 system was potentially caused by the imperfectly matched nucleotide on the 5′ end of the crRNA introduced by the U3 promoter. Therefore, we used tRNA‐crRNA to precisely produce mature crRNAs after cleaving the tRNA from the chimeric transcript (Ding *et al*., 2018). However, the tRNA did not significantly increase the mutation efficiency of STU as expected (Figure 1b). Interestingly, we found the STU‐mediated mutation with poly‐A was 1.9‐fold higher on average than that obtained with the vector lacking poly‐A. The inclusion of a poly‐A linker at the 3′ end is thought to ensure the strong expression of the Cas protein (Tang *et al*., 2016). Our results thus indicated that *Lb*Cpf1 production might be the main limitation in STU‐induced mutagenesis. To confirm the high efficiency of the STU‐poly‐A vector, two additional genomic sites located at *ALS* and *NAL* genes were targeted. Similarly, most of the regenerated plants were mutated (Figure 1b), which further indicate the high mutational efficiency in rice.

**Figure 1:**
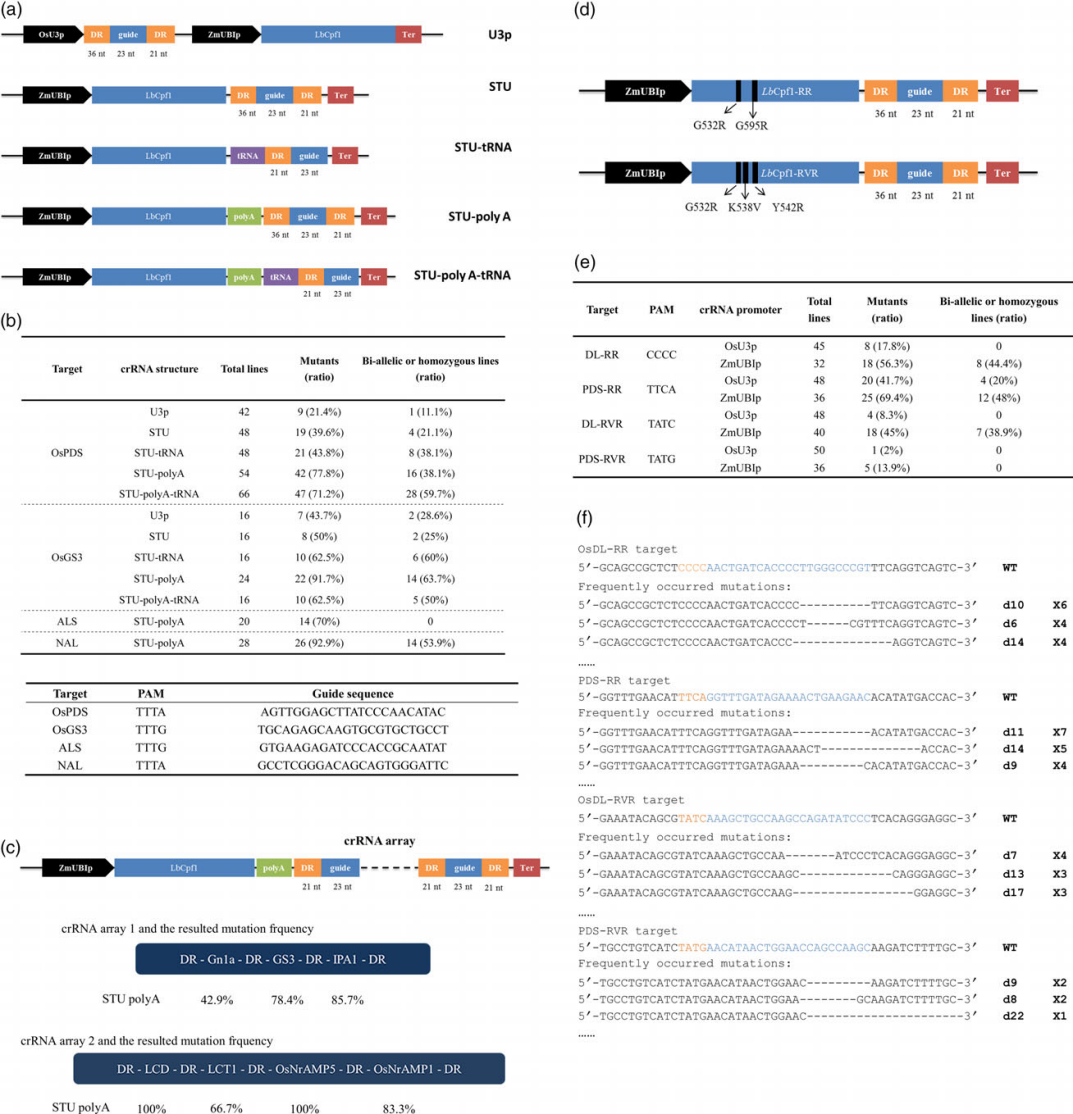
Gene editing in rice using *Lb*Cpf1in STU systems. (a) Diagrams of the CRISPR‐*Lb*Cpf1 construct. The length of the DR and guide RNA is labelled. A CaMV 35S promoter terminator (ter) was used for the *Lb*Cpf1 and the fusion transcript. (b) Upper table, targeted mutagenesis frequency induced by *Lb*Cpf1 with different crRNA expression cassettes. Plants with homozygous and biallelic mutations but not chimeric mutations were used to calculate the ratio in total mutants. Lower table, the targets examined by the *Lb*Cp1‐STU vectors. (c) Multiplexed gene editing using STU‐poly‐A vector. Upper, diagram of *Lb*Cpf1‐mediated multiplex editing construct. Lower, mutagenesis frequency of *Gn1a*‐*GS3*‐*IPA1* and *LCD1*‐*LCT1*‐*NrAMP5*‐*NrAMP1* gene arrays. (d) Schematic illustration of the expression cassettes in the engineered Cpf1 binary vector. Arrow indicates the point mutations of the specific variants. (e) Targeted mutagenesis frequency induced by *Lb*Cpf1 variants in T_0_ transgenic plants. (f) The most frequently occurring mutations induced by *Lb*Cpf1 variants in the STU plants. The top sequence comes from the WT that contains the PAM (orange) and spacer (blue) sequence. –, deleted nucleotides. The numbers of deleted nucleotides (#) are shown on the right as d#. The number on the far right indicates the plants carrying the mutation.

Cpf1‐mediated multiple‐genome editing has been achieved by expressing a crRNA array with a Pol III promoter (Wang *et al*., 2017). To determine whether a crRNA array coexpressed at the 3′‐end of a Pol II promoter‐driven *Lb*Cpf1 mRNA transcript can be used to edit multiple genomic targets in plants, two arrays comprising several guide RNAs divided by mature short direct repeats (DRs) of an *Lb*Cpf1 scaffold were tested in Japonica rice Zhonghua 11 (Figure 1c). After analysing all T_0_ plants of the *Gn1a*‐*GS3*‐*IPA1* array, 5 out of 14 lines were identified as triplet mutants. We observed that 42.9%, 78.4% and 85.7% regenerated plants were mutated at the Gn1a, GS3 and IPA1 targets respectively. Another array containing guide RNAs against four cadmium accumulation‐related genes showed even higher efficiency: all 12 regenerated lines were mutated at the *LCD* and *OsNrAMP5* loci, while 8 and 10 lines carried targeted mutations at the *LCT1* and *OsNrAMP1* sites respectively. In mammalian cells, crRNAs excised from Pol II promoter‐expressed mRNAs efficiently edited multiplexed genome targets. Very recently, two groups independently reported that coexpressing direct or intronic crRNA arrays in a single transcript with *Lb*Cpf1 or *Fn*Cpf1 has similar or higher efficiency as conventional Pol III promoter‐expressed crRNA arrays (Ding *et al*., 2018; Wang *et al*., 2018). Taken together, these results indicate that the STU strategy provides a robust and effective method for Cpf1‐mediated multiplexed genome editing in plants.

The canonical PAM of *Lb*Cpf1 is a TTTV motif, which is relatively long and results in fewer targetable sites than *Sp*Cas9. To overcome this limitation, the mutations G532R/K595R (RR) or G532R/K538V/Y542R (RVR) were made in *Lb*Cpf1 to relax the targeting scope to TYCV/CCCC or TATV PAMs in human cells (Gao *et al*., 2017). Two recent studies indicated that mutations in plant genomic targets with noncanonical PAMs could be induced by the *Lb*Cpf1 variants at variable rates (Li *et al*., 2018; Zhong *et al*., 2018). Therefore, site‐specific nucleotide substitutions were also introduced into the rice codon‐optimized *LbCpf1* in the conventional OsU3p‐expressed or improved STU vector (Figure 1d). The genome editing of plant *Lb*Cpf1‐RR and ‐RVR variants were examined at different targets in the *OsPDS* and *OsDL* genes. The STU‐expressed crRNAs showed clearly enhanced activity in generating mutations. For *Lb*Cpf1‐RR, the STU construct achieved mutation rates of 56.3% at a CCCC PAM and 69.4% at a TTCA PAM (Figure 1e), exhibiting comparable efficiency at the same PAMs sites as a system expressing crRNA in a *Lb*Cpf1‐RR‐independent cassette from the ZmUBI promoter (UBI‐crRNA; Zhong *et al*., 2018). The editing activity of *Lb*Cpf1‐RVR is relatively lower than that of the RR variant (Figure 1e). In the UBI‐crRNA system, *Lb*Cpf1‐RVR shows a preference for editing TATG PAM over TATC PAM in plant (Zhong *et al*., 2018). However, the *Lb*Cpf1‐RVR in our STU‐crRNA yielded a 45% mutation rate at the target site with a TATC PAM compared with a 13.9% rate at a TATG site (Figure 1e). These results suggested that, in addition to the PAM preference, the activity of *Lb*Cpf1‐RVR may also be determined by other factors, such as GC content and epigenetic status of the targets. Similar to previous reports, our results also confirmed that the *Lb*Cpf1 variants induced relatively large deletions at the targets, similar to *Lb*Cpf1 (Figure 1f). Notably, *Lb*Cpf1‐RR and ‐RVR variants exhibited substantially lower efficiency when using Pol III promoter‐expressed crRNA‐ribozyme cassettes (Li *et al*., 2018), further implying that the accuracy of crRNA sequence might not the principal factor affecting the activity of the CRISPR‐*Lb*Cpf1 system in plants.

Several *Lb*Cpf1 editing systems have been reported in plants, including some quite delicate designs. The mutational frequencies of these systems vary significantly, which may be due to the PAM structure, crRNA pattern, target sequence context, chromatin status or even plant transformation procedure. It will be interesting to compare the efficiency of different systems at that same genome target with exactly identical crRNA. In addition, we also noted that the efficiency at different targets may vary greatly for a single system (Ding *et al*., 2018; Wang *et al*., 2018; Zhong *et al*., 2018). We speculated that the editing activity of *Lb*Cpf1 and its derivatives may be more easily determined by the specific target. Therefore, a bioinformatics algorithm to predict robustly editable protospacers will be important for Cpf1‐mediated mutagenesis in plants. Our study indicated that crRNAs in Pol II cassettes yield higher activity than that obtained by Pol III promoters for *Lb*Cpf1 and its variants. Similarly, the high mutational frequency of *Lb*Cpf1 or *Fn*Cpf1 is normally generated in systems with the crRNA expressed from different Pol II promoters or with different transcript structure (Ding *et al*., 2018; Wang *et al*., 2018; Zhong *et al*., 2018). The results suggested that mRNA transcription and/or further processing of the crRNA is beneficial for activating the Cpf1‐crRNA complex. Overall, these studies improve the efficiency of our previous system and provide an easy‐to‐use multiplexed editing tool with extensive adaptability in the plant genome.

## Author contributions

P.W. designed the experiments and wrote the manuscript. J.Y. and P.W. supervised the research. R.X., R.Q., J.L. H.L., and Y.W. contributed to the vector construction, rice transformation, and genotyping. All authors discussed the data..

## Competing financial interests

The authors declare that they have no competing financial interests to disclose.
